# When Stability Fails: Rare Very Late Dislodgement of 3830 Lead

**DOI:** 10.1002/ccr3.71819

**Published:** 2026-01-28

**Authors:** Dilip Kumar, Niladri Ghosh, Ashesh Halder, Rabin Chakraborty, Srashti Kulshrestha, Arnab De

**Affiliations:** ^1^ Manipal Hospital EM Bypass Kolkata Kolkata India

**Keywords:** dislodgement, lumenless lead, pacemaker, pre‐syncope

## Abstract

We report a rare case of very late dislodgement of a Medtronic SelectSecure 3830 lumenless lead three years after successful left bundle branch area pacing. An 88‐year‐old patient with permanent atrial fibrillation and bifascicular block underwent VVIR pacemaker implantation in 2022 with excellent initial parameters (threshold 0.8 V at 0.5 ms, impedance 650 Ω) and stable follow‐up for three years. The patient presented with pre‐syncopal episodes, and device interrogation revealed capture failure with elevated threshold (2.25 V/0.4 ms) and decreased impedance. Fluoroscopic comparison confirmed macro‐dislodgement of the lead with coiling in the pacemaker pocket. The SelectSecure lead was successfully extracted and replaced with a conventional stylet‐driven lead positioned in the right ventricular mid‐septum. This case highlights that late lead dislodgement can occur with lumenless leads even years after successful implantation, emphasizing the need for continued vigilance during long‐term follow‐up of cardiac physiological pacing systems.

## Key Clinical Message

There is a growing interest for cardiac physiological pacing in recent times. Despite its favorable safety profile, very late lead dislodgement may occur with the Medtronic SelectSecure 3830 lumenless lead even years after implantation. We should be extremely vigilant to identify this rare complication so that restorative measures can be taken instantaneously.

## 1 Introduction

The age‐old strategy of conventional right ventricular apical pacing as backup pacing or as a part of biventricular pacing has largely been replaced by the adoption of cardiac physiological pacing (CPP) recently. With the refinement of implantation technique, operator experience and shifting trend from His‐bundle pacing towards left bundle area pacing, the procedural success has improved and the complication rates have reduced in the short and intermediate term follow‐up. Though the acute and short‐term complications pertaining to CPP remain quite obvious from various registries and case reports, the long‐term lead behavior sometimes leaves us in awe. Here we present a case of lead dislodgement 3 years after implantation of Medtronic SelectSecure 3830 lead in the left bundle branch area.

## 2 Case History

**FIGURE 1:**
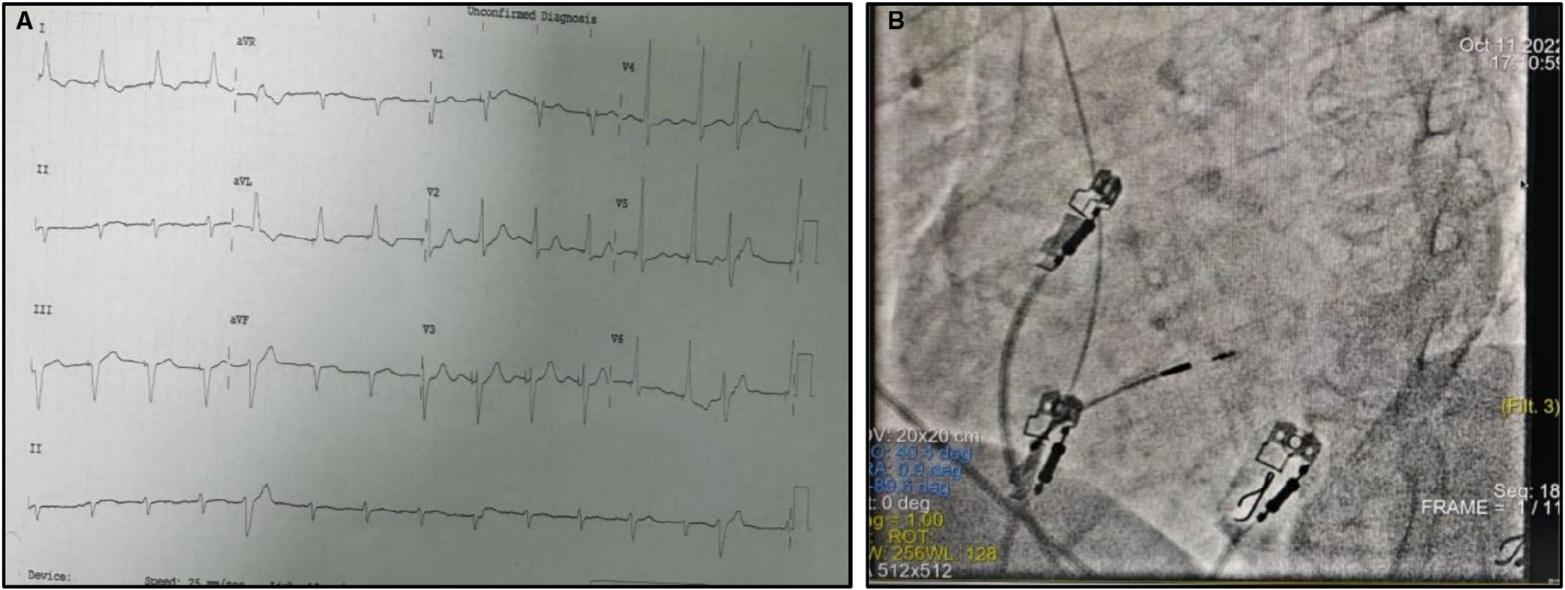
(A) ECG morphology of LBB area pacing, paced QRS in V1 is having Qr morphology with QRS duration of 110 ms (2022). (B) Medtronic SelectSecure 3830 lead location in LAO 40 degree view (2022).

An 88‐year‐old patient of permanent atrial fibrillation with slow ventricular rate and bifascicular block with recurrent syncope underwent permanent pacemaker implantation (VVIR, Vitatron G20A2 SR) in 2022. The Medtronic SelectSecure 3830 lumenless lead was positioned at the left bundle branch area with the support of the pre‐shaped C315 catheter in the first attempt. During implantation, capture of left bundle branch (LBB) was confirmed based on left ventricular activation time of 68 ms and transition from non‐selective to selective LBB area pacing (LBBAP) during threshold testing; current of injury was 15 mV. The final threshold was 0.8 V at 0.5 ms pulse width (acceptable capture threshold < 1 V/0.5 ms), lead impedance was 650 Ω (normal range 200–2000 Ω), which was re‐checked in the following day (Figure 1A,B). A Chest X‐ray was also done to look at the lead position and to exclude implantation‐related complications. This patient was doing well, and lead parameters were absolutely fine on 6‐monthly follow‐up since the implantation.

Recently he sought medical attention because of repeated pre‐syncopal attacks. Further evaluation was done to find out the etiology of pre‐syncope.

## 3 Differential Diagnosis, Investigations and Treatment

**FIGURE 2:**
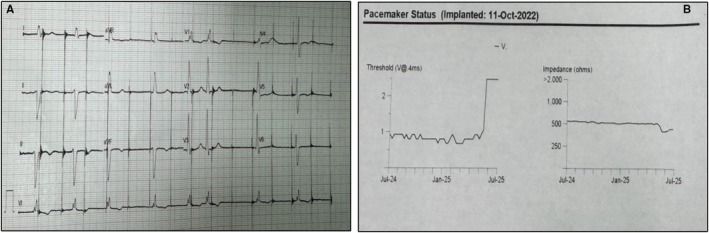
(A) ECG showed capture failure; a baseline rhythm of atrial fibrillation with bifascicular block (complete right bundle branch block with left anterior hemiblock) is seen. (B) There was a sudden increase of ventricular lead threshold, also look at the simultaneous dipping of lead impedance (pacemaker model: Vitatron G20A2 SR).

**FIGURE 3:**
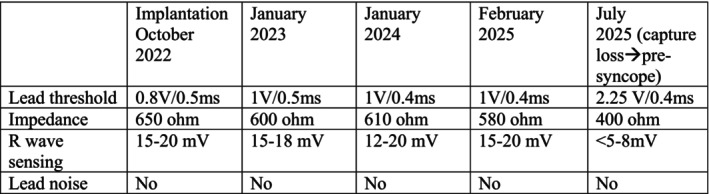
Summary of lead parameters at implantation and follow‐up visits.

The electrocardiogram showed capture failure (Figure 2A). Capture failure may happen due to several reasons, including lead or pulse generator–related problems, issues at the lead tip–myocardial interface, or underlying myocardial disease. On device interrogation it was seen that the programmed pacing output was 2 V/0.4 ms, there was an obvious sudden rise of ventricular lead threshold to 2.25 V/0.4 ms, and a sudden dip of lead impedance (though the absolute value of impedance was within normal range) was also noted. R‐wave sensing was < 5.6 to 8 mV (normal value > 5 mV) (Figures 2B and 3). No evidence of lead noise was observed; the abrupt occurrence of lead threshold and impedance change concurrently indicates a lead‐related malfunction.

**FIGURE 4:**
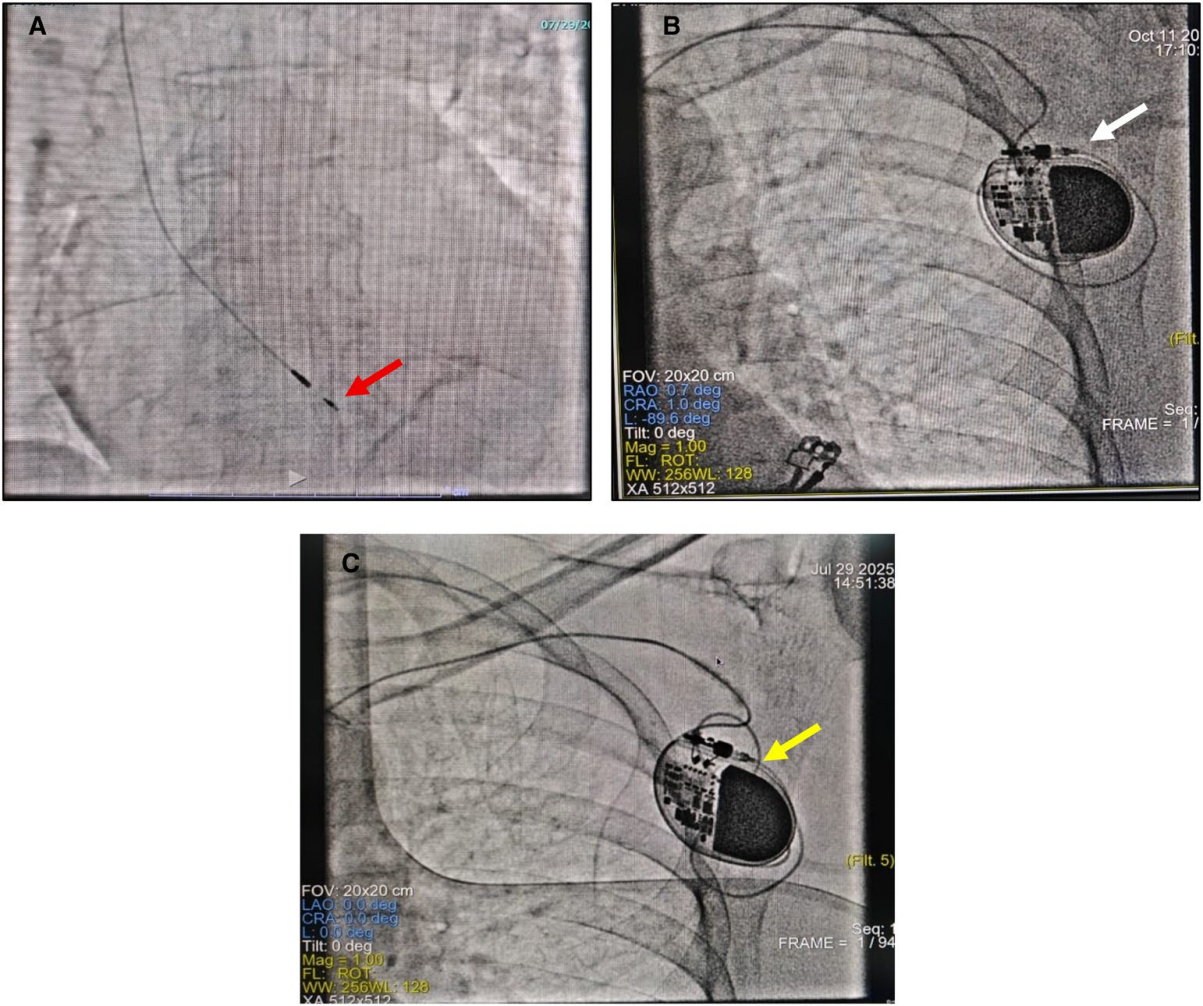
(A) Fluoroscopic picture of Medtronic 3830 lead dislodgement in 2025 (lead tip is shown in red arrow). (B) Position of pulse generator and the lead looping (white arrow) inside the pocket in 2022. (C) Position of pulse generator and the lead looping (white arrow) inside the pocket after lead dislodgement in 2025.

We reviewed the lead position fluoroscopically and compared it with the fluoroscopic picture taken during the first implantation, which suggested macro‐dislodgement of the lead (Figure 4A–C). We also noted coiling of the lead in the pacemaker pocket without gross displacement of the pulse generator or lead twisting. Afterwards, we manually extracted the SelectSecure 3830 lead after dissecting and separating the lead from surrounding fibrous tissue in and around the infraclavicular subcutaneous pocket. A new conventional 58 cm stylet‐driven lead was positioned at the right ventricular mid‐septum and connected with the old generator, ensuring that the lead parameters (threshold, impedance, R wave sensing, etc.) are within normal range.

## 4 Conclusion

This case highlighted that capture failure can occur years after device implantation with the SelectSecure 3830 (Medtronic) lumenless lead, even after ensuring adequate and satisfactory septal penetration during implantation.

## 5 Discussion

Capture failure is defined as when a pacing artifact is not followed by evidence of depolarization of the myocardium. This might happen because of a number of reasons and present at a different point in time that is, within hours to years after device implantation. Lead dislodgement or perforation, air‐bubble in the header are the reasons for capture failure seen commonly within hours to weeks of device implantation; but when it happens after years of implantation, then it is extremely important to analyze every component of the pacing system. Pulse generator issues like premature battery depletion and improper programming of pacing output may lead to capture failure. Lead‐related problems like fracture, breach of insulation, and dislodgement also result in loss of capture. Exit block at the lead‐myocardial interface and myocardial diseases because of cardiomyopathy or ischaemia may also result in a rise in threshold and capture failure. Non‐cardiac causes like electrolyte imbalance (like hyperkalemia), acidosis, recent addition or change of anti‐arrhythmic medication and recent history of MRI scan or surgery or radiofrequency ablation might contribute to alteration of threshold [1]. Though all these complications are quite well established with stylet‐driven lead but the long‐term lead behavior of the SelectSecure 3830 lumenless lead is not very well understood. In the multicentre European MELOS study LBB area pacing (LBBAP) lead dislodgement was 1.7% and the threshold rise to > 2 V was < 1% within a mean follow‐up of 6.4 months [2].

In our case, we observed capture failure associated with a rise in lead threshold and concurrent fall of impedance, with fluoroscopic evidence of lead dislodgement. The chance of having an exit block in the current scenario was not considered because of gross macro dislodgement of lead; also, we did not consider insulation breach, as the likelihood of it is less as the absolute impedance was within normal range. We also excluded other reasons of capture failure, like absence of dyselectrolytemia, ischaemia or cardiomyopathy. It is very unusual to develop lead dislodgement 3 years after the device implantation. After comparing with the previous fluoroscopic picture, we speculated that fibrosis, adhesion of the lead to the surrounding tissue and the anatomical tortuosity of the left innominate vein and its junction with the right superior vena cava in the elderly might have played an important role in late dislodgement with a possible contribution from inappropriate ipsilateral arm movement of the elderly gentleman. We observed increased lead looping inside the pocket without any obvious rotation of the pulse generator or lead twisting. This is unlike Twiddler syndrome or Reels syndrome; it is worth mentioning that our case mimics Ratchet syndrome from mechanistic point of view, but the important difference is that, unlike in the classical Ratchet syndrome our patient presented very late [3].

There are reports of late lead dislodgement of SelectSecure 3830 in children where the extra loop inside the right atrium was insufficient to match the linear growth of the children [4]. Our case indicated that it is not impossible to find very late lead dislodgement with lumenless leads irrespective of age group.

Meticulous and stepwise assessment is essential for establishing the diagnosis of capture failure so that the corrective measures can be taken appropriately. Briefly, any symptom of bradycardia (like pre‐syncope or syncope) after pacemaker implantation requires the urgent need to evaluate the integrity of the pacemaker‐lead assembly. An initial ECG finding of capture failure followed by comprehensive device interrogation will enable us to identify the problem precisely. Adjunctive imaging modality may provide further insight in specific scenarios like lead fracture or Twiddler syndrome. Based on the collective information, immediate restorative measures should be taken to minimize the adverse outcome.

## Author Contributions

**Dilip Kumar:** writing – original draft, writing – review and editing. **Niladri Ghosh:** writing – original draft, writing – review and editing. **Ashesh Halder:** resources, supervision, writing – review and editing. **Rabin Chakraborty:** supervision, validation, writing – review and editing. **Srashti Kulshrestha:** conceptualization, funding acquisition, investigation. **Arnab De:** supervision, validation, writing – review and editing.

## Funding

The authors have nothing to report.

## Consent

Written and informed patient consent obtained.

## Conflicts of Interest

The authors declare no conflicts of interest.

## Data Availability

Data sharing not applicable to this article as no datasets were generated or analyzed during the current study.
